# The influence of poly-drug use patterns on the association between opioid agonist treatment engagement and injecting initiation assistance

**DOI:** 10.1186/s13011-022-00470-6

**Published:** 2022-05-19

**Authors:** Stephanie A. Meyers-Pantele, Maria Luisa Mittal, Sonia Jain, Shelly Sun, Indhu Rammohan, Nadia Fairbairn, M-J Milloy, Kora DeBeck, Kanna Hayashi, Dan Werb

**Affiliations:** 1 Division of Infectious Diseases and Global Public Health, Department of Medicine, University of California San Diego, 9500 Gilman Drive, La Jolla, CA 92093-0507 USA; 2 Department of Psychology, San Diego State University, San Diego, CA USA; 3 School of Medicine, Universidad Xochicalco, Tijuana, BC Mexico; 4 Department of Family Medicine and Public Health, University of California San Diego, La Jolla, CA USA; 5 Centre on Drug Policy Evaluation, St. Michael’s Hospital, 30 Bond Street, Toronto, ON M5B 1W8 Canada; 6 British Columbia Centre on Substance Use, Vancouver, BC Canada; 7 Department of Medicine, University of British Columbia, Vancouver, BC Canada; 8 School of Public Policy, Simon Fraser University, Vancouver, BC Canada; 9 Faculty of Health Sciences, Simon Fraser University, Vancouver, BC Canada

**Keywords:** Opioid agonist treatment, Overdose prevention, HIV prevention, HCV prevention, Persons who inject drugs, Methadone, Suboxone, Treatment as prevention

## Abstract

**Background:**

Evidence suggests people who inject drugs (PWID) prescribed opioid agonist treatment (OAT) are less likely to provide injection drug use (IDU) initiation assistance. We investigated the association between OAT engagement and providing IDU initiation assistance across poly-drug use practices in Vancouver, Canada.

**Methods:**

*Preventing Injecting by Modifying Existing Responses* (PRIMER) is a prospective study seeking to identify structural interventions that reduce IDU initiation. We employed data from linked cohorts of PWID in Vancouver and extended the findings of a latent profile analysis (LPA). Multivariable logistic regression models were performed separately for the six poly-drug use LPA classes. The outcome was recently assisting others in IDU initiation; the independent variable was recent OAT engagement.

**Results:**

Among participants (*n* = 1218), 85 (7.0%) reported recently providing injection initiation assistance. When adjusting for age and sex, OAT engagement among those who reported a combination of high-frequency heroin and methamphetamine IDU and low-to-moderate-frequency prescription opioid IDU and methamphetamine non-injection drug use (NIDU) was associated with lower odds of IDU initiation assistance provision (Adjusted Odds Ratio [AOR]: 0.18, 95% CI: 0.05–0.63, *P* = 0.008). Significant associations were not detected among other LPA classes.

**Conclusions:**

Our findings extend evidence suggesting that OAT may provide a population-level protective effect on the incidence of IDU initiation and suggest that this effect may be specific among PWID who engage in high-frequency methamphetamine and opioid use. Future research should seek to longitudinally investigate potential causal pathways explaining the association between OAT and initiation assistance provision among PWID to develop tailored intervention efforts.

## Introduction

People who inject drugs (PWID) play a key role in injection drug use (IDU) initiation processes, with 68–88% of IDU initiates reporting they received education, guidance, or were directly injected by more experienced PWID during their initiation event [1–3]. PWID are also impacted by a variety of injection-related harms, including overdose, HIV, hepatitis C, and bacterial infections [4, 5]; they are also at greatest risk for these harms within the first 5 years of initiating IDU [6, 7]. Given the documented vulnerability of PWID that have recently transitioned into IDU, it is critical that research and harm reduction efforts be directed towards preventing transitions into this mode of substance use, particularly in settings like Vancouver, Canada that are disproportionately impacted by the North American overdose crisis [8–10].

Preliminary evidence suggests that PWID on opioid agonist treatment (OAT) are less likely to report initiating others into IDU [11, 12]. Specifically, PWID from both San Diego and Vancouver that reported a history of OAT had significantly decreased odds (Vancouver: Adjusted Odds Ratio [AOR] = 0.52; 95% Confidence Interval [CI] = 0.31–0.87) of providing IDU initiation assistance [11, 12]. For people who inject opioids, OAT mitigates withdrawal symptoms and effectively reduces the frequency of injecting unregulated opioids [13, 14]; we hypothesize that OAT may also reduce the frequency with which PWID expose injection-naïve individuals to IDU, thereby reducing the potential for PWID to encounter requests for injection initiation assistance. Further, it is estimated that an expansion of OAT coverage to 40%, 50%, or 60% for PWID could reduce injection initiation events by 11.5%, 17.3%, and 22.8% per annum, could potentially reduce PWID population size, and the expansion of opioid- and injection-related epidemics [15, 16]; though data on the potential impact of OAT among polydrug-using subpopulations is lacking.

Recent evidence suggests that the prevalence of methamphetamine and other poly-drug use is increasing among PWID who use opioids in North America [17–19]. This is concerning given that those who inject methamphetamine in conjunction with opioids are at greater risk for injection-related harms, including overdose, compared to PWID who inject only opioids [20]. Furthermore, methamphetamine use has been associated with IDU initiation in Vancouver and Tijuana, Mexico [21, 22], and with providing injection initiation assistance to others among PWID in Tijuana [22]. This suggests that efforts to prevent injection initiation, such as the provision of OAT to PWID who provide injection initiation assistance, should focus on specific subpopulations of PWID at highest risk. As such, the current study sought to further investigate whether previously-identified protective associations between OAT engagement and injection initiation assistance provision [12] were found across PWID with differing poly-drug use practices in Vancouver, Canada.

## Methods

### Study design

The *PReventing Injecting by Modifying Existing Responses* (PRIMER) study seeks to assess the impact of socio-structural factors on the risk that PWID provide injection initiation assistance [3]. The PRIMER study protocol and rationale have been previously described [3]. For the present analysis, data were drawn from three linked open prospective cohort studies of people who use drugs (PWUD) in Vancouver, Canada: The *At-Risk Youth Study* (ARYS; street-involved youth aged 14–26 who use drugs), the *AIDS Care Cohort to evaluate Exposure to Survival Services* study (ACCESS; HIV-seropositive adult [≥18 years of age] PWUD), and the *Vancouver Injection Drug Users Study* (VIDUS; HIV-seronegative adult PWID). To be eligible, participants had to report unregulated use of (i.e., illegal/street-based) drugs other than cannabis ≤30 days prior to baseline. The PRIMER baseline is defined as the visit when specific survey items soliciting reports of assisting others in their first injection were introduced into the cohort questionnaires (December, 2014-May, 2017). The current study is cross-sectional and was restricted to participants who reported past six-month IDU at PRIMER baseline. The inclusion criterion of recent IDU was selected given that the provision of IDU initiation assistance is a relatively rare event [23, 24], and that focusing on the population most likely to provide this assistance would allow for the most accurate description of the phenomena of interest [1, 3]. This study was approved by Institutional Review Boards at UC San Diego and the University of British Columbia-Providence Health Care. All participants provided written informed consent.

### Measures

Participants completed an interviewer-administered questionnaire assessing sociodemographics, drug use behaviors, health services enrollment, and other domains. The primary outcome, recently assisting others in their first injection, was assessed via the following survey item: “*In the past six months, have you helped someone inject who had never injected before?*”). The independent variable, recent OAT engagement, was defined via endorsement of the statement: “*In the past six months have you been in* (*methadone/methadose program or Suboxon*e) *treatment*?” The covariates of age and sex (i.e., male/female) were selected based on previous studies of IDU initiation assistance [22–24], and all variables of interest were captured at participants’ PRIMER baseline visit. Analyses were stratified based on drug use practices identified within a previously conducted latent profile analysis (LPA) ([25]; Rammohan I, Jain S, Sun X, Marks C, Milloy M-J, Hayashi K, et al: Identifying latent polydrug use patterns and assessing their association with the provision of injection initiation assistance in three north American settings: a latent profile analysis, In Preparation). The LPA methods have previously described in full; briefly, six classes of poly-drug use were identified based on participants’ recent IDU and non-injection (NIDU) drug type (i.e., methamphetamine, heroin, cocaine, and prescription opioids [PO]) and frequency of use (i.e., low, moderate, and high) ([25]; Rammohan I, Jain S, Sun X, Marks C, Milloy M-J, Hayashi K, et al: Identifying latent polydrug use patterns and assessing their association with the provision of injection initiation assistance in three north American settings: a latent profile analysis, In Preparation).

### Statistical analyses

Cross-sectional bivariate associations between OAT engagement and providing IDU initiation assistance were assessed among each LPA class using cross-tabulation and Fisher’s exact tests. Multivariable logistic regression models were performed for each LPA class separately to study the independent association of reporting OAT use on the provision of injection initiation assistance while controlling for age and sex. Analyses were performed using *R* software (version 3.5.1). Two-sided *P*-values < 0.05 were considered statistically significant.

## Results

Among 1218 participants, 162 participants (13.3%) were in Class 1, defined by past six-month high-frequency methamphetamine IDU and low-to-moderate NIDU (Table 1). Additionally, 128 (10.5%) participants were in Class 2, defined by moderate-to-high-frequency heroin IDU, high-frequency heroin NIDU, and moderate-to-high-frequency methamphetamine IDU and NIDU. Nearly a quarter (*n* = 295; 24.2%) were in Class 3, defined by low-frequency heroin, methamphetamine, cocaine, and PO IDU and NIDU. Seventeen percent of participants (*n* = 201; 16.5%) were in Class 4, defined by high-frequency heroin and methamphetamine IDU, low-to-moderate-frequency PO IDU, and methamphetamine NIDU. Further, 279 (22.9%) were in Class 5, defined by high-frequency heroin IDU. Lastly, 153 (12.6%) participants were in Class 6, defined by high-frequency cocaine IDU and low-moderate heroin IDU. The proportion reporting recent OAT engagement was 51.9% in the overall sample and was 30.9% (Class 1), 51.6% (Class 2), 53.9% (Class 3), 47.3% (Class 4), 62.4% (Class 5), and 58.2% (Class 6) among each class, respectively. The proportion reporting providing recent injecting initiation assistance in the overall sample was (7.0%) and 9.9% (Class 1), 12.5% (Class 2), 2.0% (Class 3), 11.4% (Class 4), 5.7% (Class 5), and 5.2% (Class 6) in each group, respectively.

**Table 1:** Association between OAT and Provision of Injecting Initiation Assistance among PWID subgroups in Vancouver, Canada (*n* = 1218)

	Did not assist others in their first injection in the past 6 months	Assisted others in their first injection in the past 6 months	Total	*P*-value^a^
**1**. High Frequency Methamphetamine IDU; Low-to-Moderate NIDU^b^				
OAT=No	98 (87.5%)	14 (12.5%)	112 (69.1%)	0.152
OAT=Yes	4 (96.0%)	2 (4.0%)	50 (30.9%)	
Total	146 (90.1%)	16 (9.9%)	162	
**2**. Moderate-to-High Frequency Heroin IDU; High Frequency Heroin NIDU; and Moderate-to-High Frequency Methamphetamine IDU and NIDU^b^				
OAT=No	55 (88.7%)	7 (11.3%)	62 (48.4%)	0.792
OAT=Yes	57 (86.4%)	9 (13.6%)	66 (51.6%)	
Total	112 (87.5%)	16 (12.5%)	128	
**3**. Low Frequency Use of All Drugs^b^				
OAT=No	133 (97.8%)	3 (2.2%)	136 (46.1%)	> 0.999
OAT=Yes	156 (98.1%)	3 (1.9%)	159 (53.9%)	
Total	289 (98.0%)	6 (2.0%)	295	
**4**. High Frequency Heroin IDU and Methamphetamine IDU, and Low-to-Moderate Frequency PO IDU and Methamphetamine NIDU^b^				
OAT=No	86 (81.1%)	20 (18.9%)	106 (52.7%)	**< 0.001**
OAT=Yes	92 (96.8%)	3 (3.2%)	95 (47.3%)	
Total	178 (88.6%)	23 (11.4%)	201	
**5**. High Frequency Heroin IDU^b^				
OAT=No	96 (91.4%)	9 (8.6%)	105 (27.7%)	0.121
OAT=Yes	167 (96.0%)	7 (4.0%)	274 (72.3%)	
Total	263 (94.3%)	16 (5.7%)	379	
**6.** High Frequency Cocaine IDU and Low-to-Moderate Heroin IDU^b^				
OAT=No	61 (95.3%)	3 (4.7%)	64 (41.8%)	> 0.999
OAT=Yes	84 (94.4%)	5 (5.6%)	89 (58.2%)	
Total	145 (94.8%)	8 (5.2%)	153	

^a^Fisher’s exact test

^b^The class refers to activities during the previous six months

*IDU* Injection Drug Use, *NIDU* Non-Injection Drug Use, *OAT* Opioid Agonist Treatment

We conducted six separate multivariable regression models for each LPA-identified poly-drug use class with age and sex included as covariates (Table 2). Among a subset of participants characterized by high-frequency heroin and methamphetamine IDU, low-to-moderate-frequency PO IDU, and methamphetamine NIDU (Class 4), participants who reported recent OAT engagement had significantly lower odds of reporting recent injection initiation assistance (AOR: 0.18, 95% CI: 0.05–0.63, *P* = 0.008). The association between OAT engagement and injection initiation assistance was not significant among other LPA classes.

**Table 2:** Multivariable logistic regression models assessing the association between assisting others in their first injection and OAT engagement in Vancouver, Canada

Variable	AOR	(95% CI)	*P*-value
**1. High Frequency Methamphetamine IDU; Low to Moderate NIDU**^**a**^ **(*n*** **= 162)**			
OAT engagement^a^	0.34	(0.07–1.60)	0.173
Age	0.97	(0.92–1.02)	0.224
Male sex	4.64	(0.99–21.65)	0.051
**2. Moderate-to-High Frequency Heroin IDU; High Frequency Heroin NIDU; Moderate-to-High Frequency Methamphetamine IDU and NIDU**^a^ **(*n*** **= 127)**			
OAT engagement^a^	2.04	(0.64–6.49)	0.230
Age	0.94	(0.87–1.01)	0.094
Male sex	0.64	(0.21–1.93)	0.427
**3. Low Frequency Use of All Drugs**^a^ **(*n*** **= 295)**			
OAT engagement^a^	1.71	(0.29–10.26)	0.557
Age	0.91	(0.84–0.98)	**0.014**
Male sex	1.25	(0.21–7.40)	0.808
**4. High Frequency Heroin IDU and Methamphetamine IDU; Low-to-Moderate Frequency PO IDU and Methamphetamine NIDU**^a^ **(*n*** **= 199)**			
OAT engagement^a^	0.18	(0.05–0.63)	**0.008**
Age	0.96	(0.91–1.01)	0.105
Male sex	1.89	(0.68–5.23)	0.222
**5. High Frequency Heroin IDU**^a^ **(*n*** **= 279)**			
OAT engagement^a^	0.46	(0.16–1.32)	0.148
Age	0.93	(0.89–0.98)	**0.003**
Male sex	1.37	(0.47–3.95)	0.565
**6. High Frequency Cocaine IDU; Low-to-Moderate Heroin IDU**^a^ **(*n*** **= 153)**			
OAT engagement^a^	1.02	(0.22–4.79)	0.978
Age	0.90	(0.83–0.97)	**0.005**
Male sex	0.42	(0.08–2.16)	0.299

^a^The variable refers to activities during the previous six months

*AOR* Adjusted Odds Ratio, *95% CI* 95% Confidence Interval, *IDU* Injection Drug Use, *NIDU* Non-Injection Drug Use, *OAT* Opioid Agonist Treatment

## Discussion

We found a protective association against providing injection initiation assistance among PWID who were recently enrolled in OAT and who had recently engaged in high-frequency heroin and methamphetamine IDU, low-to-moderate-frequency PO IDU, and methamphetamine NIDU, but not among those reporting other poly-drug use patterns. This expands our understanding of the benefits of OAT with respect to the risk of providing injection initiation assistance [11–14]. While methamphetamine IDU has been associated with providing initiation assistance among PWID [22], our findings imply that the potential protective effect of OAT on initiation assistance provision may be heightened among PWID who inject methamphetamines in conjunction with heroin and PO.

We did not find significant associations between OAT and providing initiation assistance among those reporting methamphetamine and heroin IDU, without PO, or among those reporting high-frequency cocaine IDU and low-to-moderate heroin IDU. These findings could potentially reflect limited statistical power to detect associations within some classes given the small size of some subgroups. To that end, they highlight the need for future research to more fully investigate OAT engagement among PWID with varying poly-drug use patterns. Nevertheless, our findings extend previous research demonstrating that people enrolled in OAT and who use cocaine are more likely to report continued cocaine and heroin use when accessing these services compared to those who use other substances [26, 27]. The current findings potentially indicate that alternative therapeutic approaches (e.g., buprenorphine), OAT dosing strategies, or tailored OAT engagement supports are needed to meet the needs of individuals who engage in complex poly-drug use [28].

Previous research indicates that PWID who report injecting in the presence of injection-naïve individuals are more likely to be asked to provide initiation assistance [1], and that acquiescing to requests often occurs within withdrawal and economic need contexts [29]. Though we found no association between OAT engagement and providing initiation assistance among those who solely engaged in high frequency heroin IDU, past research has also found higher proportions of PWID who perceive their OAT dosage to be “too low” reported assisting others compared to those who perceived an “adequate” or “high” dosage [12]. As such, if OAT alleviates experiences of withdrawal, it could buffer against requests for initiation for experienced PWID [30]. This further suggests that PWID who report methamphetamine, heroin, and PO IDU could benefit from OAT provision and supports to reduce PWID engagement in IDU initiation events. However, recent evidence from Vancouver demonstrates that methamphetamine use is associated with OAT discontinuation [31], which complicates efforts to retain those at highest risk of providing initiation assistance. The current study indicates, however, that achieving retention among the subpopulation of PWID reporting methamphetamine and opioid (e.g., heroin and PO) IDU may yield a secondary community-level preventive benefit by potentially reducing injection initiation assistance incidence.

### Limitations

This study has limitations typical of observational cross-sectional research. Non-probability sampling was used for participant recruitment due to the mobile nature of the population and the corresponding difficulty of developing a sampling frame. Consequently, we cannot assume generalizability for the population of PWID in this study setting. Secondly, we relied on self-report, and underreporting of experiences of initiating others into injecting is likely given it is highly stigmatized, particularly within the context of an overdose crisis [32]; however, research suggests that PWID accurately self-report OAT engagement [33] and substance use-related behaviors [34], indicating these measures are valid. Additionally, to protect against potential response bias, participants were notified of the confidential nature of the study. Third, given the cross-sectional nature of the data collected and the fact that we did not construct causal inference models, we cannot determine causality among OAT engagement and reductions in providing IDU initiation assistance. Further, given that we conducted separate multivariable analyses for each LPA class, we cannot directly compare findings between classes. Finally, we were limited in the number of covariates we could include in our multivariable models due to small subsample sizes and because our outcome was a rare event. Small subsample sizes could have also increased the likelihood of committing Type II error and potentially limited the statistical significance of the study findings [35]. Nevertheless, the findings of this study are in-line with similar studies that found associations between substance use, OAT engagement, and injection initiation events [11, 22].

## Conclusion

These findings suggest that, beyond OAT’s effectiveness in managing opioid use disorder, it may also have a secondary protective effect on the expansion of IDU among high-risk populations, including those engaging in high frequency polysubstance use (i.e., methamphetamine, heroin, and PO in combination). This further indicates the need to assess whether OAT service expansion among PWID populations who concurrently inject multiple substances, including methamphetamine and opioids, may reduce the incidence of IDU initiation. These efforts could have the potential to limit transitions into drug injecting, and consequently reduce injection-related harms, including overdose and blood-borne disease transmission.

## Data Availability

The datasets used and/or analyzed during the current study are available from the corresponding author upon reasonable request.
